# Potential Use of a Combined Bacteriophage–Probiotic Sanitation System to Control Microbial Contamination and AMR in Healthcare Settings: A Pre-Post Intervention Study

**DOI:** 10.3390/ijms24076535

**Published:** 2023-03-31

**Authors:** Maria D’Accolti, Irene Soffritti, Francesca Bini, Eleonora Mazziga, Luca Arnoldo, Antonella Volta, Matteo Bisi, Paola Antonioli, Patrizia Laurenti, Walter Ricciardi, Sara Vincenti, Sante Mazzacane, Elisabetta Caselli

**Affiliations:** 1 Section of Microbiology, Department of Chemical, Pharmaceutical and Agricultural Sciences, and LTTA, University of Ferrara, 44121 Ferrara, Italy; maria.daccolti@unife.it (M.D.);; 2 CIAS Research Center, University of Ferrara, 44122 Ferrara, Italy; 3 Department of Medicine, University of Udine, 33100 Udine, Italy; 4 Department of Infection Prevention Control and Risk Management, S. Anna University Hospital, 44124 Ferrara, Italy; 5 Department of Health Sciences and Public Health, Section of Hygiene, Catholic University of the Sacred Heart, 00168 Rome, Italy; 6 Department of Woman and Child Health and Public Health, Fondazione Policlinico Universitario A. Gemelli IRCCS, 00168 Rome, Italy

**Keywords:** AMR, bioburden, HAI, probiotics, bacteriophages, sanitation

## Abstract

Microbial contamination in the hospital environment is a major concern for public health, since it significantly contributes to the onset of healthcare-associated infections (HAIs), which are further complicated by the alarming level of antimicrobial resistance (AMR) of HAI-associated pathogens. Chemical disinfection to control bioburden has a temporary effect and can favor the selection of resistant pathogens, as observed during the COVID-19 pandemic. Instead, probiotic-based sanitation (probiotic cleaning hygiene system, PCHS) was reported to stably abate pathogens, AMR, and HAIs. PCHS action is not rapid nor specific, being based on competitive exclusion, but the addition of lytic bacteriophages that quickly and specifically kill selected bacteria was shown to improve PCHS effectiveness. This study aimed to investigate the effect of such combined probiotic–phage sanitation (PCHSφ) in two Italian hospitals, targeting staphylococcal contamination. The results showed that PCHSφ could provide a significantly higher removal of staphylococci, including resistant strains, compared with disinfectants (−76%, *p* < 0.05) and PCHS alone (−50%, *p* < 0.05). Extraordinary sporadic chlorine disinfection appeared compatible with PCHSφ, while frequent routine chlorine usage inactivated the probiotic/phage components, preventing PCHSφ action. The collected data highlight the potential of a biological sanitation for better control of the infectious risk in healthcare facilities, without worsening pollution and AMR concerns.

## 1. Introduction

It is now recognized that built environments (BEs) can be considered as superorganisms with their own microbiome similar to those observed in living organisms [1]. Notably, compared with unconfined natural environments, the microbiome of more confined environments shows reduced biodiversity and increased antimicrobial resistance (AMR), being composed essentially of microbes of human origin growing under the selective pressure exerted by continuous disinfection [2]. BE microbiomes and human occupants represent a complex system characterized by mutual influencing interactions, as the composition of the microbiome can have an important impact on humans’ health and people in turn can contribute to its origin and composition [3]. In the hospital environment, these features appear particularly important as the persistent contamination by human pathogens can contribute to the onset of the so-called healthcare-associated infections (HAIs), which represent a major concern for hospitalized people [4], affecting over four million people per year in the European Community, and directly causing over 37,000 deaths, of which around 10,000 are in Italy [5,6]. Indeed, the hospital environment represents a reservoir of pathogens, spread by hospitalized patients, sanitary staff, and visiting people [7,8]. These pathogens, under the selective pressure exerted by the massive and continuous use of disinfectants and antimicrobial drugs, frequently become resistant to several antimicrobials (multi-drug resistant, MDR), complicating the HAI therapy and further worsening the risk associated with the acquisition of a HAI [9].

Consistent with this, surveillance programs and improvements in hygiene practices are introduced worldwide to prevent AMR and HAI spread [10], including conventional chemical-based sanitation of the environment, which has been further significantly increased recently to counteract the spread of the pandemic Severe Acute Respiratory Syndrome Coronavirus type 2 (SARS-CoV-2) virus [11]. However, most chemical sanitizers have a high environmental impact, temporary action [12,13], and a recognized role in the potential selection of resistant strains [14], while also eliminating potentially beneficial microbes [15,16,17].

In the search for innovative and eco-friendly sanitation strategies able to overcome these concerns, we previously studied a probiotic cleaning hygiene system (PCHS) based on the use of an eco-friendly detergent with the addition of selected spores of the probiotic *Bacillus* genus, showing that it could provide effective control of pathogenic bacteria, fungi, and enveloped viruses (including SARS-CoV-2), while simultaneously controlling and decreasing the AMR diffusion and the associated HAIs [13,17,18,19,20,21,22,23]. Based on a competitive exclusion mechanism, PCHS provides a gradual shaping of the environment microbiome, needing around two weeks to achieve a stable microbiome balance [22]. These features render PCHS unsuitable for providing rapid decontamination as could be needed during emergency situations. In addition, PCHS action is not specific, and this may represent another limitation when a rapid counteraction against specific pathogens is desired (for example, in the case of epidemic outbreaks, or in rooms hosting patients colonized by specific bacterial strains).

To improve PCHS rapidity and specificity features, we considered the addition of lytic bacteriophages, based on their reported usage as environmental decontaminants [24,25,26]. Bacteriophages are procaryotic viruses characterized by a very narrow host range and a rapid action, and thanks to these features, have been suggested as effective agents for biological control against foodborne and plant pathogens, and for decontamination of industrial surfaces, farm facilities, and wastewater [24,25,26,27,28,29,30,31,32,33]. Notably, they are also active against HAI-associated pathogens persistently contaminating hospital surfaces, including MDR bacteria such as *Pseudomonas aeruginosa* [34], and methicillin-resistant *Staphylococcus aureus* (MRSA) [35]. Nevertheless, the potential use of lytic phages as environmental sanitizers in hospitals has been rarely investigated in the field, despite the promising results obtained in a few published studies that report the effective use of aerosolized phage cocktails for room terminal cleaning as an efficient procedure to control carbapenem-resistant *Acinetobacter baumannii* (CRAB) contamination and associated infections [36,37]. One study by us showed that phages could effectively eliminate several bacteria isolated from hospitals, including Gram-positive, Gram-negative, and MDR strains, and that they could be added to PCHS without losing activity [38]. Moreover, in a small pilot study, we reported their ability to improve the action of PCHS, promoting its stabilizing capability [39]. Based on these data, the present study aimed to assess the effectiveness of a combined probiotic–phages system (PCHSφ) in two large Italian hospitals, comparing its effect with that obtained with chemical-based and PCHS-based sanitation. As a proof of concept, the different sanitation procedures were tested in the General Medicine wards, targeting the contamination by *Staphylococcus* spp. in the bathroom areas, as staphylococcal contamination is the most prevalent in hospitals and bathrooms are the most contaminated areas.

## 2. Results

### 2.1. Study Set-Up

**Figure 1:**
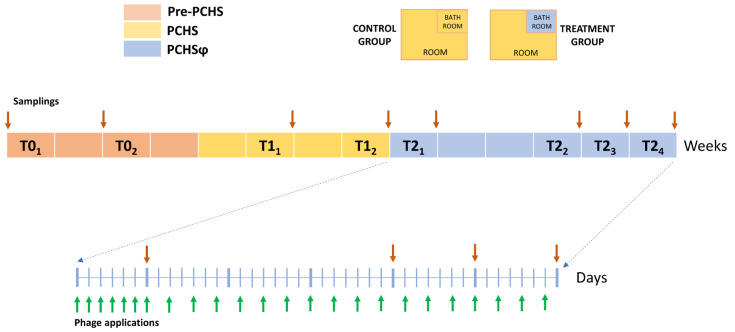
Study design. The study period (14 weeks) was subdivided in three phases: the pre-PCHS T0 period (red; four weeks), during which wards received conventional chemical sanitation; the PCHS T1 period (yellow; four weeks), during which PCHS replaced chemical sanitation; and the PCHSφ T2 period (blue; six weeks), during which phages were added to PCHS sanitation in the bathrooms of 12/24 randomly selected rooms (treatment group), while the remaining rooms received only PCHS without phages (control group). Phage applications (green arrows) and samplings (red arrows) are indicated.

The applicability and effectiveness of a combined probiotic–phage system of sanitation was tested in the General Medicine wards of two large Italian hospitals. The study lasted 14 weeks during the COVID-19 pandemic (March–June 2021) and included three phases: (1) the pre-PCHS period, during which hospitals maintained the conventional chemical-based sanitation (T0); (2) the PCHS period, during which PCHS replaced chemical disinfection (T1); and (3) the PCHSφ period, during which phages were added to PCHS (T2). The added phages were directed against *Staphylococcus* spp. and applied in the rooms’ bathrooms, based on previous data showing that staphylococcal contamination is the most prevalent in the hospital environment and the bathrooms the most contaminated areas in hospitals [22]. Extraordinary disinfection with 3% chlorine was allowed during the whole study in cases of confirmed COVID-19 hosted in the enrolled wards. Environmental samplings were performed during the whole study period to monitor the microbial contamination on treated surfaces. The study design and timing are schematized in Figure 1.

### 2.2. Phage Susceptibility of Staphylococcus spp. Hospital Isolates

Prior to introducing PCHS and PCHSφ sanitations, the surface samples collected in the pre-PCHS period were used to isolate, enumerate, and identify the *Staphylococcus* species present in the enrolled hospital settings to assess their amount and susceptibility to phage killing. The results evidenced a high prevalence of *Staphylococcus* spp. in the sampled surfaces, including mostly coagulase-negative strains and less than 10% of coagulase-positive *S. aureus* species, with no significant differences between the enrolled hospitals. Antimicrobial susceptibility tests performed on all *S. aureus* isolates showed that 72% of isolates were MDR, as judged from the resistance to methicillin and at least four other antibiotics (not shown), with no significant differences between the two enrolled hospitals.

**Figure 2:**
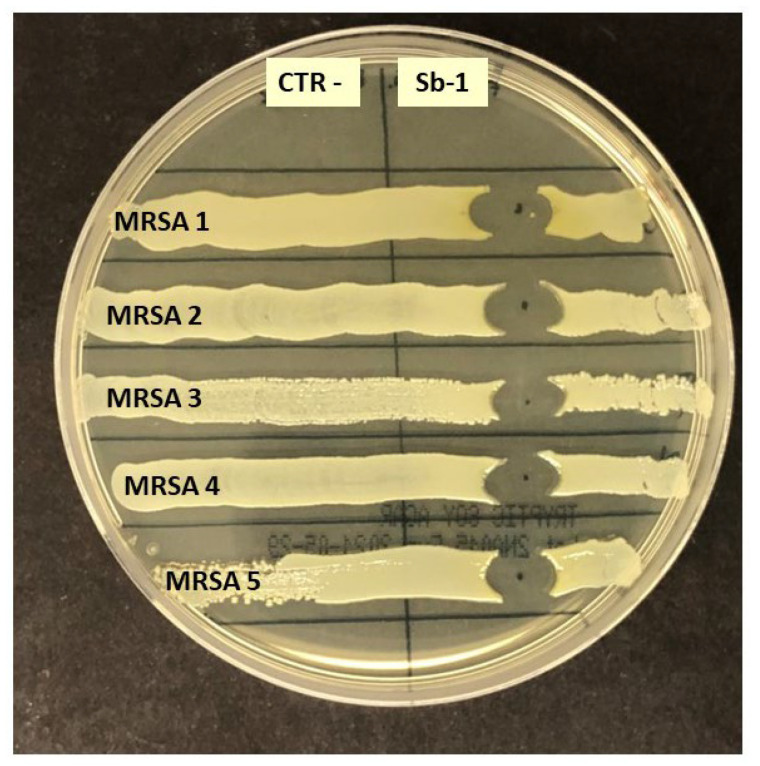
Phage susceptibility test of MRSA isolated from hospital surfaces in the pre-PCHS period. Each isolate was assessed for Sb-1 phage susceptibility by the spot test assay. CTR-, negative control (TSB); Sb-1, phage suspension. The results shown are for five representative MRSA isolates.

All the MDR isolates were tested by spot agar assay for their susceptibility to the Staphylococcal bacteriophage Sb-1 preparation, already used in previous studies for its wide-range killing ability [39]. The results confirmed that Sb-1 phages were capable of killing both MDR *S. aureus* and coagulase-negative isolates (Figure 2). Based on previous optimization tests, the Sb-1 phage preparation was added to PCHS to obtain a multiplicity of infection corresponding to 1000:1 (phage:bacteria ratio) that was previously shown to be optimal for surface treatment [39].

### 2.3. Impact of PCHS and PCHSφ Sanitation on Microbial Contamination

Eight environmental samplings were performed to monitor the level of six groups of important HAI-associated pathogens, including *Staphylococcus* spp. (specifically targeted by the PCHSφ treatment), *Enterobacteriaceae* spp., *Pseudomonas* spp., *Clostridium difficile*, *Enterococcus* spp., and fungi (*Candida* and *Aspergillus* spp.). Since extraordinary 3% chlorine disinfection was allowed during the whole study period, based on the detection of COVID-19 cases in the ward, the number of chemical disinfection interventions was also monitored. The results showed a more frequent use of chlorine disinfection in HS-2, compared with the HS-1 center (Table 1), with 6 and 2 interventions in the T1 period, and 17 and 3 interventions in the T2 period, in the HS-2 and HS-1 centers, respectively.

**Figure 3:**
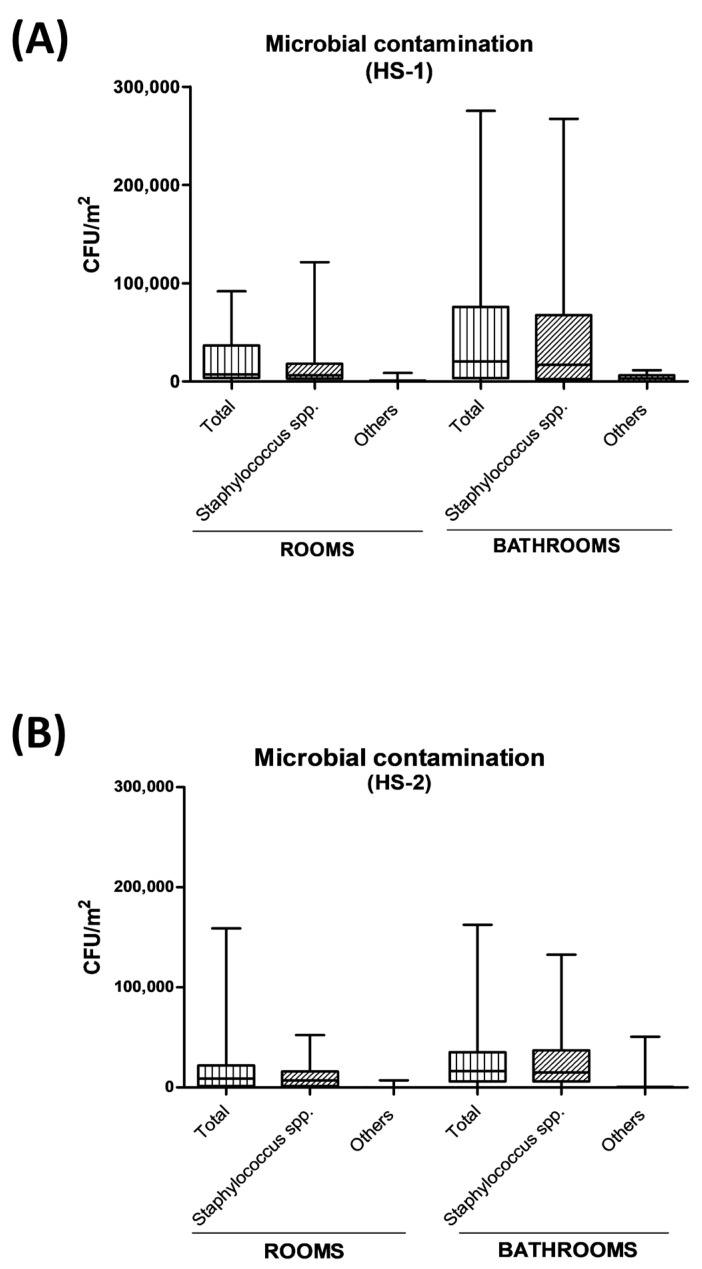
Total and staphylococcal contamination in the enrolled hospital wards at T0 (pre-PCHS period). Total contamination is expressed as the sum of *Staphylococcus* spp., *Enterobacteriaceae* spp., *Pseudomonas* spp., *Clostridium* spp., *Aspergillus* spp., and *Candida* spp. CFU counts. Samplings were performed in rooms (floor, bed footboard) and bathrooms (floor, sink, shower plate). The results are expressed as CFU number per m^2^; median values (lower part of the box) and Q3 values (upper part of the box, representing the 75% percentile values) are shown, together with min. and max. values. (**A**) HS-1. (**B**) HS-2.

The results of microbial monitoring performed in the pre-PCHS period showed the presence in both hospitals of a persistent surface contamination by clinically relevant pathogens, particularly prominent in the bathrooms areas, confirming the observations of previous studies [18,22,39]. In detail (Figure 3), the total contamination detected in rooms and bathrooms of HS-1, expressed as the sum of the searched pathogens, corresponded to a median value of 7158 CFU/m^2^ in rooms (range 0–91,789) and 20,211 CFU/m^2^ in bathrooms (range 421–275,368 CFU/m^2^). Of those, *Staphylococcus* spp. represented up to 88% of the total detected pathogens, corresponding to 6316 CFU/m^2^ in rooms (range 0–121,311 CFU/m^2^) and to 17,053 CFU/m^2^ in bathrooms (median value, range 0–215,398 CFU/m^2^). The other pathogens accounted for 842 CFU/m^2^ in rooms (median value, range 0–8751 CFU/m^2^) and 3158 CFU/m^2^ in bathrooms (median value, range 0–11,523 CFU/m^2^). Similarly, in the HS-2 hospital, the total contamination was 8790 CFU/m^2^ in rooms (range 0–158,900 CFU/m^2^) and 16,420 CFU/m^2^ in bathrooms (range 0–162,526 CFU/m^2^) (Figure 3). In addition, *Staphylococcus* spp. represented up to 91% of the total detected pathogens, with 7921 CFU/m^2^ in rooms (median value, range 0–52,211 CFU/m^2)^ and 14,948 CFU/m^2^ in bathrooms (median value, range 0–132,632 CFU/m^2^). The other detected pathogens amounted to 869 CFU/m^2^ in rooms (median value, range 0–7158 CFU/m^2^), and 1472 CFU/m^2^ in bathrooms (median value, range 0–50,631 CFU/m^2^).

**Figure 4:**
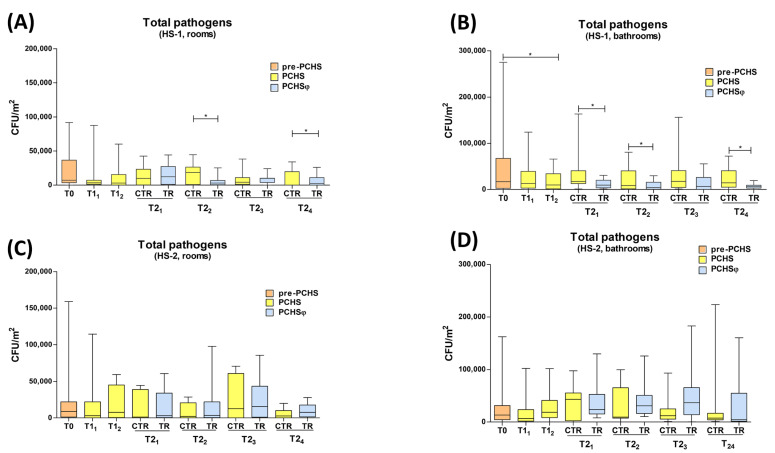
Total pathogen contamination in enrolled hospitals. The sanitation types applied in the different study periods are identified by colors: red (chemical-based sanitation), yellow (PCHS) and blue (PCHSφ). (**A**) Rooms of HS-1. (**B**) Bathrooms of HS-1. (**C**) Rooms of HS-2. (**D**) Bathrooms of HS-2. All the results are expressed as CFU number per m^2^; Median values (lower part of the box) and Q3 values (upper part of the box, representing the 75% percentile values) are shown, together with min. and max. values. Sampling times: T0 (chemical-based sanitation in all enrolled bathrooms), T1 (PCHS in all enrolled bathrooms), and T2 (PCHS and PCHSφ in control and treated bathrooms, respectively). *, *p* < 0.05.

With the introduction of PCHS to replace chemical disinfection (PCHS period), a reduction in surface pathogens was observed in both healthcare settings (Figure 4). Specifically, in HS-1 (Figure 4A,B), two weeks after PCHS implementation, (T1_1_) total surface pathogens diminished to 3368 CFU/m^2^ in rooms (range 0–87,579 CFU/m^2^) and 13,264 CFU/m^2^ (range 0–124,211 CFU/m^2^) in bathrooms, corresponding to decreases of 52.9% and 34.4% in rooms and bathrooms, respectively. At T1_2_ (corresponding to four weeks of PCHS usage), the count of pathogens amounted to 2947 CFU/m^2^ in rooms (range 0–60,211 CFU/m^2^) and 9895 CFU/m^2^ (range 0–66,105 CFU/m^2^) in bathrooms, with a decrease of 58.8% in rooms and 51% in bathrooms, which were statistically significant differences compared with T0 (*p* < 0.05). During the T1 period, no differences were observed between the level of contamination found in the bathroom assigned to continue receiving PCHS alone (control group) and in those assigned to receiving the combined PCHSφ sanitation (treated group). Following the addition of anti-staphylococcal phages in the bathrooms of the twelve selected rooms (T2 period), significant differences were detected in the contamination level depending on the type of sanitation applied. Specifically, at T2_1_, total pathogens corresponded to 17,684 CFU/m^2^ (median value, range 1263–163,368 CFU/m^2^) in the PCHS, and 9684 CFU/m^2^ (median value, range 1263–30,737 CFU/m^2^) in the PCHSφ group of bathrooms (−45.2%; *p* < 0.05). At T2_2_, the median pathogen load was 8632 CFU/m^2^ in the PCHS control group (range 0–80,842 CFU/m^2^) and 4211 CFU/m^2^ in the PCHSφ treated group (range 0–29,895 CFU/m^2^) (−51.2%; *p* < 0.05). At T2_3_, the respective values were 17,895 CFU/m^2^ (median, range 0–156,632 CFU/m^2^) and 6737 CFU/m^2^ (median, range 0–55,579 CFU/m^2^) (−62.3%). At T2_4_, the median values detected were 14,737 CFU/m^2^ (range 0–19,368 CFU/m^2^) and 6106 ± CFU/m^2^ (range 0–19,398 CFU/m^2^) in the PCHS and PCHSφ groups, respectively (−58.6%). A slightly larger decrease in microbial contamination was also observed in the rooms whose ensuite bathrooms were treated with PCHSφ, compared with those receiving only PCHS sanitation in both bathroom and room spaces (Figure 4A). The detected differences were statistically significant at the T2_2_ and T2_4_ timepoints.

In contrast, in the HS-2 hospital, no significant decrease was observed in total surface contamination during the whole study period, despite some non-significant decreases in the median and maximum values observed at T1_1_ at two weeks after PCHS introduction when the total pathogen CFU were 3158 CFU/m^2^ in rooms and 6948 CFU/m^2^ in bathrooms (median values, range 0–114,526 and 0–102,316 CFU/m^2^) (Figure 4C,D). Instead, at T1_2_ and at subsequent timepoints, the contamination level increased or decreased inconsistently and non-significantly in both sampled areas, independently of the type of sanitation applied.

**Figure 5:**
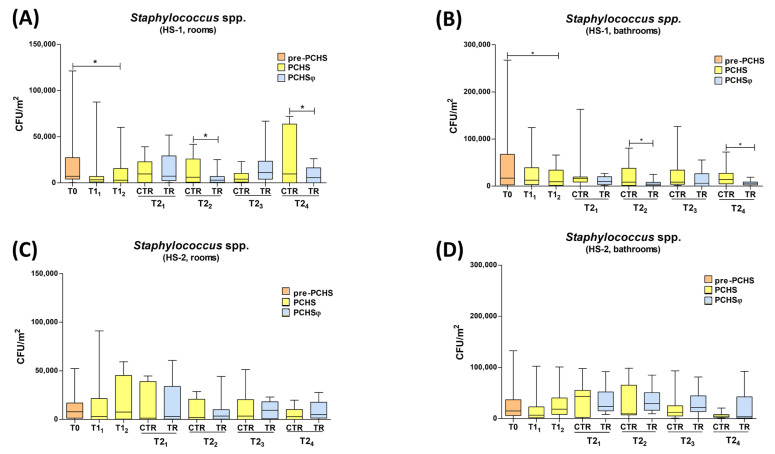
Staphylococcal contamination in enrolled hospitals. The sanitation types applied in the different study periods are identified by colors: red (chemical-based sanitation), yellow (PCHS) and blue (PCHSφ). (**A**) Rooms of HS-1. (**B**) Bathrooms of HS-1. (**C**) Rooms of HS-2. (**D**) Bathrooms of HS-2. All the results are expressed as CFU number per m^2^; Median values (lower part of the box) and Q3 values (upper part of the box, representing the 75% percentile values) are shown, together with min. and max. values. Sampling times: T0 (chemical-based sanitation in all enrolled bathrooms), T1 (PCHS in all enrolled bathrooms), and T2 (PCHS and PCHSφ in control and treated bathrooms, respectively). *, *p* < 0.05.

Consistent with the staphylococcal prevalence in the sampled areas, the decrease in staphylococci CFUs accounted for most of the microbial decrease observed upon PCHS and PCHSφ usage (Figure 5).

Specifically, in HS-1 rooms (Figure 5A), staphylococci median level decreased to 3368 CFU/m^2^ at T1_1_ (range 0–87,159 CFU/m^2^), and to 2947 CFU/m^2^ at T1_2_ (median value, range 0–60,211 CFU/m^2^) (*p* < 0.05). During the PCHSφ period (T2_1_–T2_4_), the staphylococci median level was 9685 CFU/m^2^ (range 0–39,158 CFU/m^2^) in the rooms of the control group, and 7305 CFU/m^2^ (range 421–51,789 CFU/m^2^) in those of the TR group, suggesting that PCHSφ sanitation in the bathrooms could also influence the room’s contamination. At T2_2_, the differences between CTR and TR groups were maintained, showing 6105 and 2947 CFU/m^2^ (median values, range 0–41,684 and 0–25,263 CFU/m^2^) in the CTR and TR groups, respectively. At T2_3_, staphylococcal counts were instead 4000 CFU/m^2^ in the CTR group (range 842–23,158 CFU/m^2^) and 11,158 CFU/m^2^ (range 0–66,947 CFU/m^2^) in the TR group. At the final timepoint (T2_4_), staphylococci counts were 9684 CFU/m^2^ in the CTR group (median value, range 0–72,000 CFU/m^2^) and 5684 CFU/m^2^ (range 0–26,105 CFU/m^2^) in the TR group (*p* < 0.05).

In the bathroom areas (Figure 5B), staphylococcal counts decreased as well, compared with T0, showing 12,632 CFU/m^2^ at T1_1_ (range 0–124,211 CFU/m^2^) and 9263 CFU/m^2^ at T1_2_ (range 0–66,105 CFU/m^2^). In the PCHSφ period (T2_1_-T2_4_), clear differences between the CTR and TR groups emerged, showing a significantly larger decreases in staphylococci in bathrooms receiving PCHSφ compared with those receiving PCHS alone, at all times tested. At T2_1_, the median staphylococci level corresponded to 16,421 CFU/m^2^ (range 0–162,947 CFU/m^2^) in CTR and 9684 CFU/m^2^ (range 1263–26,947 CFU/m^2^) in the TR group, showing a further −41% decrease compared with PCHS alone, although the difference was not statistically significant. At T2_2_, *Staphylococcus* spp. were 8632 CFU/m^2^ (median value, range 0–80,842 CFU/m^2^) and 4000 CFU/m^2^ (range 0–24,842 CFU/m^2^) in the CTR and TR groups, respectively (−53.6%, *p* < 0.05); at T2_3_, staphylococci median counts were 8421 CFU/m^2^ in the CTR group (range 1263–126,737 CFU/m^2^) and 6105 CFU/m^2^ in the TR group (range 0–55,579 CFU/m^2^) (−27.5%); and at T2_4_, they were 14,316 CFU/m^2^ (range 0–72,421 CFU/m^2^) and 6105 CFU/m^2^ (range 0–18,947 CFU/m^2^) (−57.4%, *p* < 0.05) in the CTR and TR groups, respectively.

In contrast, but consistent with the results observed for total contamination, in the HS-2 setting, the staphylococcal contamination showed an inconsistent trend, with a slight but not significant decrease in staphylococci observed only at T1_1_ (median value 6948 CFU/m^2^, range 0–102,316 CFU/m^2^), whereas no reduction in *Staphylococcus* CFUs was observed at later times, neither in rooms nor in bathrooms (Figure 5C,D).

The main results obtained for staphylococcal contamination by applying PCHS and PCHSφ sanitations are summarized in Table 2.

### 2.4. Assessment of PCHS-Bacillus and Sb-1 Phage on Treated Hospital Surfaces

**Figure 6:**
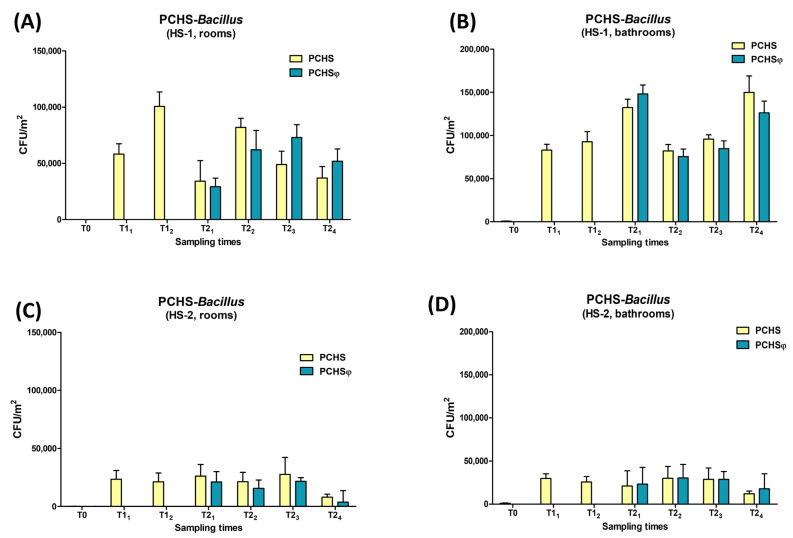
Amounts of PCHS-*Bacillus* in the enrolled hospital wards. (**A**) Rooms of HS-1. (**B**) Bathrooms of HS-1. (**C**) Rooms of HS-2. (**D**) Bathrooms of HS-2. All the results are expressed as mean CFU number per m^2^ ± S.D. values. Sampling times: T0 (chemical-based sanitation in all enrolled bathrooms), T1–T2 (PCHS in all enrolled bathrooms), and T3–T6 (PCHS and PCHSφ in control and treated bathrooms, respectively).

All the collected surface samples were also analyzed for the presence of PCHS-derived *Bacillus* probiotics and anti-staphylococcal Sb-1 phages on surfaces, by *Bacillus* CFU count and specific qPCR targeted to detect and quantify Sb-1 genome, respectively, as previously described [22,39]. The results showed that, compared with the values detected at T0 (pre-PCHS period), the *Bacillus* CFUs increased significantly in the PCHS-period (T1) as expected (Figure 6). The increase was observed in all PCHS-treated areas, including both rooms and bathrooms, with no significant differences between the two types of areas. However, while in the HS-1 setting, the *Bacillus* CFUs peaked around 100,000 and 150,000 CFU/m^2^ in the treated rooms and bathrooms, respectively, in the HS-2 setting, the *Bacillus* count did not exceed 30,000 CFU/m^2^ for any tested surface. During the subsequent PCHSφ period (T2), the number of *Bacillus* CFUs did not vary between bathrooms receiving PCHS alone or PCHSφ, showing that the addition of phages had no impact on the ability of *Bacillus* probiotics to colonize treated surfaces.

**Figure 7:**
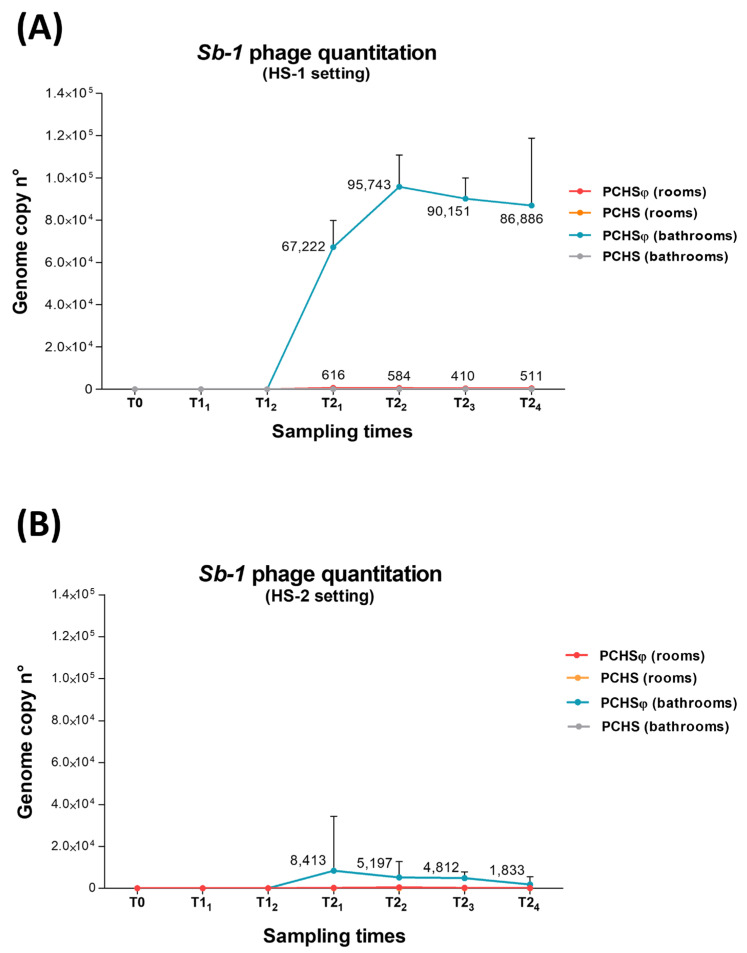
Anti-*Staphylococcus* Sb-1 phage DNA quantification in treated areas. (**A**) HS-1 setting. (**B**) HS-2 setting. Results are expressed as mean genome copy number per surface sample (corresponding to 100 cm^2^) ± S.D. values. Mean values obtained in positive samples are also shown in labels.

Similarly, the testing for the Sb-1 phage genome on treated surfaces evidenced the presence of phage DNA in PCHSφ-treated bathrooms, whereas the control bathrooms sanitized with PCHS alone did not display any phage presence (Figure 7). However, the phage load was substantially different in the two enrolled hospitals, with that detected in HS-1 (Figure 7A) around 1 Log higher compared with the load detected in HS-2 (Figure 7B). Moreover, while in HS-1 the phage titer was increasing during the T2 period (in particular, at the early T2_1_ and T2_2_ timepoints), a definite decrease was instead observed in the HS-2 setting in the same period. Some phage genomes were also detectable in the rooms whose bathrooms received PCHSφ sanitation, compared with the other sampled areas not receiving PCHSφ (Figure 7A), suggesting some passive transport of the phages from the bathroom to the room area.

### 2.5. Impact of PCHS and PCHSφ Sanitation on AMR

**Figure 8:**
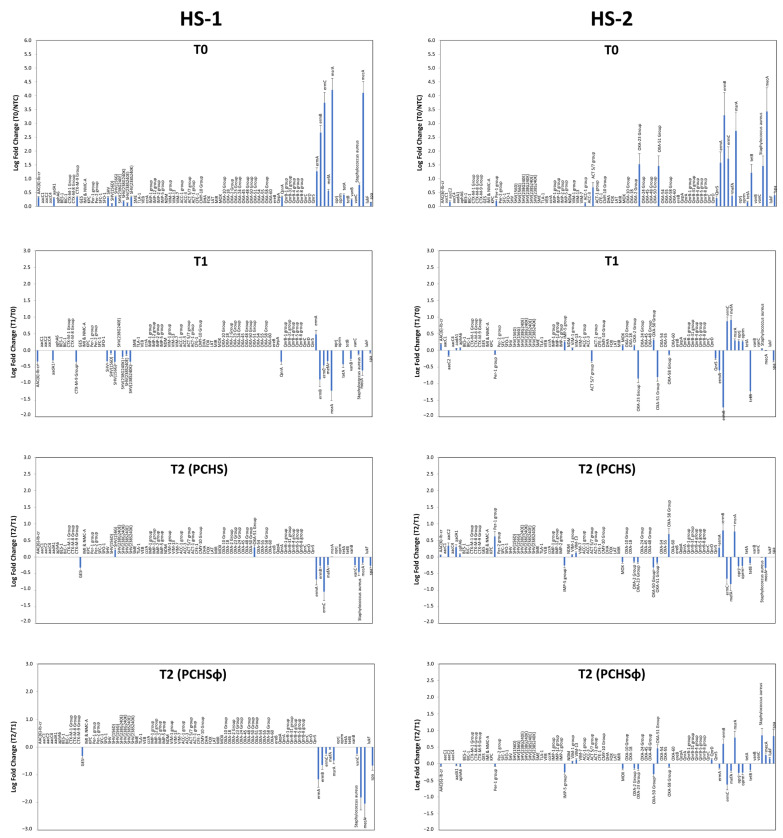
Resistome characterization of hospital surface microbiome. Left panels, HS-1. Right panels, HS-2. T0, R genes detected at basal level during the pre-PCHS period. T1, R genes detected during the PCHS period (T1_2_ sampling). T2, R genes detected during the PCHSφ period (T2_2_ sampling). Results are expressed as mean values of Log_10_ fold change compared with the respective controls, represented by negative controls (NTC) at T0, T0 values for T1 results, and T1 values for T2 results. Results refer to duplicate samples in two independent assays.

The effect of the different types of sanitation on AMR prevalence in the hospital microbiome was assessed by using a qPCR microarray able to simultaneously detect and quantify 84 resistance (R) genes. The analysis was performed on surface samples collected from both bathrooms and rooms at T0 (pre-PCHS period), T1_2_ (as indicative of the PCHS period), and T2_2_ (as indicative of the PCHSφ period). The results obtained at T0 showed, as expected, that the sampled surfaces in both hospitals hosted a microbial population harboring several R genes conferring resistance against macrolides, methicillin, and class-C/class-D β-lactamases (Figure 8), with small variations between HS-1 and HS-2 (Table 1). In detail, the most prevalent R genes of the HS-1 microbiome, in order of abundance, were *msrA*, *mecA*, *ermC*, *ermB*, and *ermA*, followed by lower but detectable levels of *Aac (6)-Ib-cr*, *aadA1*, *CTX-M-9 Group*, *SHV*, *SHV (156G)*, *SHV (238S240K)*, *mefA*, *qnrA*, *tetA* and *vanB* genes. Similarly, the most prevalent R genes detected in the HS-2 microbiome were, in order of abundance, *mecA*, *ermB*, *msrA*, *ermC*, *ermA*, *tetB*, *OXA-23* and *OXA-51 Groups*, followed by lower but detectable levels of *ACT 5/7 group*, *AAcC2*, *Per-1 group*, *QnrS*, *AacC2*, and *mefA* genes.

The detected genes conferred resistance against most classes of antibiotics, such as beta-lactams (including methicillin), carbapenems, macrolides, aminoglycosides, tetracyclines, and fluoroquinolones. *S. aureus* and its virulence gene *spa* were also detected in both HS-1 and HS-2 settings. The detected R genes, their activity against drug type, and bacterial species known to harbor most frequently the detected genes are reported in Table 3.

No significant differences were detected in bathroom and room microbial populations as to the amount of detected R genes, although some of them appeared slightly more abundant in bathrooms compared with rooms.

Following PCHS introduction, the picture substantially changed in the two hospitals (T1_2_). In HS-1, a decrease in all R genes was observed compared with T0, which was maintained and strengthened at T2_2_ with the addition of anti-staphylococcal phages (PCHSφ). This was associated with a further significant decrease in *S. aureus* and *spa* gene compared with PCHS alone (*p* < 0.01). Conversely, in HS-2 a decrease in some R genes was observed at T1_2_, including *ermB*, *tetB*, *OXA-23 Group*, *OXA-51 Group* and *spa* genes. However, some R genes were instead increased, including *msrA*, *oprj*, and *oprm*. At T2_2_, no differences were observed in PCHSφ-treated compared with PCHS-treated areas. Rather, increases in *S. aureus* and the *spa* gene were observed in both groups, accompanied by small but detectable increases in *msrA*, *mefA*, *OXA-58*, and other R genes (Figure 8).

## 3. Discussion

The persistent contamination of hospital surfaces by clinically relevant pathogens displaying AMR features is recognized as a major concern for public health, significantly contributing to difficult-to-treat HAI onset. Thus, a stable and specific decontamination strategy would be highly desirable to prevent infectious risk for hospitalized patients. Till now, such a control of microbial bioburden has been addressed using conventional chemical disinfection, which was massively introduced during the COVID-19 pandemic, even in non-sanitary environments, to counteract the spread of SARS-CoV-2. As most disinfectants are associated with the potential selection of resistant strains [14], the massive and continuous use of chemical disinfectants has further worsened the AMR spread and concern [11,40,41,42,43,44,45]. In contrast, we recently showed that a probiotic-based sanitation (PCHS, probiotic cleaning hygiene system) could stably decrease surface pathogens and revert their AMR content, ultimately reducing the HAI incidence [18,19,23]. These effects were moreover associated with a consistent decrease in the HAI therapy costs, due to the decreased HAI number and to the possibility of using less expensive antibiotics [19,46]. However, PCHS action is gradual and non-specific, being based on competitive exclusion; thus, it does not appear adequate to manage specific outbreaks in a short time. Toward these goals, lytic bacteriophages were instead recently proposed, as they can rapidly attack specific bacterial targets without altering the whole microbiome [47]. We recently reported that they can be effective against most MDR hospital isolates and can be added to PCHS without losing their activity [38,39]. Thus, here we analyzed the feasibility and effectiveness of phage addition to PCHS sanitation in a pre-post intervention study in two large Italian hospitals located in Ferrara (HS-1) and Rome (HS-2). The study aimed to investigate the potential of such a system in effectively counteracting *Staphylococcus* species, one of the most prevalent MDR contaminants of the hospital environment responsible for most HAIs. Phage decontamination was applied to bathroom areas as they represent one of the main contaminated areas in the hospital environment. Staphylococcal contamination was assessed throughout the study period, lasting 14 weeks, together with contamination by other clinically relevant pathogens and their AMR. In parallel, the presence of applied PCHS probiotics and anti-staphylococcal phages on treated surfaces was constantly monitored.

In accordance with our previous data, the *Staphylococcus* genus was the most prevalent group in the hospital surface microbiome at the beginning of the study (T0, pre-PCHS period), and the anti-staphylococcal Sb-1 phage preparation confirmed its wide range ability to kill all the collected MDR isolates, even those displaying MDR features [38].

As hypothesized, the results obtained in one of the two centers (HS-1) showed a significant decrease in staphylococcal and pathogen contamination upon PCHS introduction, compared with T0, and the reduction further increased with the addition of anti-staphylococcal Sb-1 phages to PCHS (PCHSφ period), confirming previous results [38]. Accordingly, the resistome analyses of the contaminating population at T0 provided evidence for the presence of several R genes conferring resistance against different classes of antibiotics, including methicillin, macrolides, and B-lactams. All the R genes detected at T0 significantly decreased when PCHS was introduced in place of chemical disinfection, and the reduction was further increased by the addition of phages, thanks to their capacity for eliminating *Staphylococcus* species, highlighting the potential of these anti-bacterial agents to effectively control specific pathogen targets in the hospital environment. However, in the second enrolled hospital (HS-2), the results were substantially different, as no significant decreases in staphylococcal contamination and/or R genes abundance were observed upon introduction of PCHS and PCHSφ sanitations.

In the attempt to elucidate the reasons for such a different trend, we analyzed the presence and number of PCHS-*Bacillus* probiotics and Sb-1 phages on treated surfaces, revealing a completely different situation in the two enrolled centers. Namely, whereas in HS-1, the amounts of both probiotics and phages were high as expected based on their concentration in the used solutions and preparations, in HS-2, the increases in both biological agents were significantly lower than expected. In short, while probiotic *Bacillus* were close to 10^5^ CFU/m^2^ for the whole PCHS and PCHSφ periods in HS-1, their number did not exceed 3 × 10^4^ CFU/m^2^ in HS-2, a value detected only at T1_2_ and T2_2_ timepoints, which is substantially lower than that needed to be effective. Similarly, Sb-1 phages were detected at a roughly constant amounts in the HS-1 PCHSφ treated areas, corresponding to about 9 × 10^5^ genome copies per m^2^, but in HS-2, they were ten times lower, reaching a concentration of 8 × 10^4^ genome copies per m^2^ at T3 but decreasing at the following times.

The technical procedures used for PCHS and PCHSφ application were identical in the two enrolled hospitals, and both hospitals had trained professional cleaning staff, whereas the number of emergency disinfection interventions was very different in HS-1 and HS-2. In fact, when examining the number of extraordinary 3% NaClO disinfections performed in the study period, a clear-cut difference emerges in the frequency of such disinfections, with the HS-2 setting showing a very frequent usage of this procedure compared with the sporadic use of emergency disinfection in HS-1. This is crucial, since while PCHS is compatible with some disinfectants (including ethanol) [48], we already observed in previous studies that the effect of PCHS was reversed by the simultaneous daily use of bactericidal/sporicidal chemical disinfection [49]. This is related to the mechanism of PCHS action, which is exerted by the vegetative forms of the *Bacillus* spores contained in the PCHS product that germinate on treated surfaces [20,22,23]. Such vegetative forms can effectively compete with pathogens, gradually eliminating and replacing them thanks to metabolic and proliferative advantage, and to the production of antimicrobial compounds (bacteriocins) [20]. Furthermore, the inactivation of phages and their genomes because of prolonged chemical exposure has been documented [50,51]. The lack of modulating action by probiotics and decrease in specific killing by lytic phages may have thus likely favored the recontamination processes, facilitating the increase in pathogens toward pre-PCHS levels. This condition also may explain the lack of decrease in the drug-resistance gene determinants in HS-2, compared with the decrease observed in HS-1. In fact, despite starting from a very similar picture in terms of AMR types (R genes) and amounts in the two enrolled centers, a significant abatement of the R determinants detected at T0 was only observed in the HS-1 center.

Taken together, the collected data confirm the higher effectiveness of PCHS in comparison with conventional chemical disinfection, and show that the addition of specific phages to PCHS can provide a targeted and larger decrease in the specific bacterial target on treated surfaces, making it possible to counteract rapidly the outbreak of specific hospital pathogens that are potentially responsible for difficult-to-treat HAIs and the worst patient outcomes. Moreover, in consideration of the high environmental impact of chemical disinfectants, such a biological approach also appears as a promising “green” alternative to the massive usage of disinfectants. Notably, this study also shows that a simultaneous massive use of sporicidal disinfectants may nullify the potential PCHS/PCHSφ effectiveness by causing inactivation of its biological agents. Rather, since high-level disinfectants are needed in specific circumstances, more precise management and timing of disinfectant application could allow the continued action of probiotics and bacteriophages and ensure the maintenance of PCHS/PCHSφ effectiveness.

The main limitations of the study include the low number of enrolled hospitals and the short time period of PCHS and PCHSφ application, due to the restrictions imposed by the COVID-19 regulations at the time of the study. A higher number of healthcare settings would make it possible to obtain more generalizable data, and an extended study could be useful to evaluate the PCHSφ impact on HAI incidence caused by different specific bacterial targets, as recently reported for some Intensive Care Unit infections [36]. Last, an assessment of any eventual development of phage resistance should also be included in future studies lasting longer periods to ascertain the potential for the development of phage-resistant bacteria.

## 4. Materials and Methods

### 4.1. Study Settings

A pre-post interventional study was performed in two large Italian public hospitals, including the Fondazione Policlinico A. Gemelli IRCCS (Rome, Italy), and the University Hospital of Ferrara (Ferrara, Italy), after approval by the Local Ethics Committees and authorization of the Hospital Medical Directors (for Ferrara, document n° 0009760 of 20 March 2021; for Rome, approval by the Hospital Infections Committee, document n° 0019959/21 of 19 February 2021, registered on 31 May 2021). For the study, Ferrara hospital was denominated Healthcare Setting n.1 (HS-1), and Rome hospital was denominated Healthcare Setting n.2 (HS-2). One entire General Medicine ward was enrolled in each hospital, with superimposable features, each including twenty-four rooms equipped with an internal bathroom. Room and bathroom surfaces had identical internal dimensions, with bathroom measuring about 4.5 m^2^. The study was performed during the COVID-19 pandemic and lasted 14 weeks in both enrolled hospitals (from 22 March 2021 to 28 June 2021).

### 4.2. Study Design and Sanitation Procedures

At the time of enrolment, both HS-1 and HS-2 were routinely sanitized by the chemical-based mandatory protocol imposed by the Italian Ministry of Health to manage the COVID-19 pandemic [52]. The study included three phases: (1) a 4-week period during which hospitals maintained the conventional chemical-based sanitizing procedures (T0, pre-PCHS period); (2) a 4-week period during which PCHS replaced the chemical sanitation (T1, PCHS period); (3) a final 6-week period during which specific anti-staphylococcal phage decontamination was added to PCHS (T2, PCHSφ period). In the last PCHSφ period, the enrolled ward rooms were randomly subdivided into two groups, with 12/24 rooms receiving only PCHS sanitation in their bathrooms (control group, CTR) and 12/24 receiving combined PCHS/phage sanitation in their bathrooms (treated group, TR). The room environment, excluding the bathroom area, continued to be sanitized by PCHS alone in both CTR and TR groups.

All sanitizing procedures were performed daily in both enrolled hospitals by trained staff in the early morning. Specifically, PCHS is a registered sanitation system (PCHS^®^, Copma, Ferrara, Italy) and was applied by specific mops, as previously described [19,38,39]; phage application was performed by nebulization, as previously described [39]. In detail, the “Staphylococcal bacteriophage, Sb-1” containing 10^10^ PFU/mL (Eliava Institute, Tbilisi, Georgia) was used for phage treatment. The phage concentrate was diluted in the PCHS detergent previously prepared at a working dilution of 1:100 in water and subsequently filtered with 0.45 µm filters to remove the probiotic cells to avoid an excess of probiotics in the PCHSφ treated areas compared with areas receiving only PCHS. A final concentration of 3–6 × 10^8^ PFU per bathroom was used, corresponding to a multiplicity of infection (M.O.I.) phage:bacteria of 1000:1. The nebulization of the phage suspension was performed by using volume and time of application to ensure a homogeneous distribution and persistence of phages on the treated surfaces, as determined in previous studies [38,39]. Specifically, 0.5 L of suspension was nebulized in each bathroom for 4 min, a time allowing persistence of an aqueous layer on treated surfaces for 10 min, which was proven to be the time necessary and sufficient to permit efficient contact between phages and bacterial targets [38,39]. Phage application was performed daily for 1 week (7 applications in 7 days), and then it was applied on alternate days (17 applications in 34 days) for the remaining study phase. Extraordinary disinfection interventions based on the usage of 3% chlorine were allowed when confirmed COVID-19 cases were hosted in the enrolled wards.

### 4.3. Environmental Sampling

Eight environmental sampling campaigns were performed during the whole study period: two samplings during the pre-PCHS phase (T0_1_ and T0_2_, with one week interval), two samplings during the PCHS phase (T1_1_ and T1_2_ at 2 and 4 weeks after PCHS introduction, respectively), and four samplings during the PCHS-plus phase (T2_1_, T2_2_, T2_3_, and T2_4_ at 1, 4, 5, and 6 weeks after phage decontamination introduction, respectively). Each environmental sampling was performed 7 h after sanitation [12,19] at five points per room, including three points in the bathroom (bathroom floor, bathroom sink, and bathroom shower plate), and two points in the adjacent room (floor and bed footboard). Sampling was performed by two different methods depending on the following type of analysis. For microbiological analyses, samples were collected in duplicate using replicate organism detection and counting (RODAC) plates containing general or specific media. Specifically, the following media were used: Tryptic soy agar (TSA, Sharlab, Milan Italy) for the total count; Baird Parker agar (Sharlab, Milan, Italy) for *Staphylococcus* spp. (including *Staphylococcus aureus*) detection and *Bacillus* count; cetrimide agar (Sharlab, Milan, Italy) for *Pseudomonas* spp. detection; MacConkey agar (Sharlab, Milan, Italy), selective for *Enterobacteriaceae*; Herella agar (Lickson, Palermo, Italy), selective for *Acinetobacter* spp.; *Clostridium difficile* selective agar for *Clostridium difficile* growth (Lickson, Palermo, Italy); Bile Esculin Agar (BEA) (Incofar, Modena, Italy) selective for *Enterococcus* spp.; and Sabouraud dextrose agar (Liofilchem, Millipore, Milan, Italy) for mycetes (including *Aspergillus* spp., *Candida albicans*) detection.

For the molecular analyses, the same points were sampled in duplicate by sterile rayon swabs rubbed on a 100 cm^2^ surface, as previously described [16,19,39]. The swabs were then put in 5 mL of TSB broth (Biolife, Monza, Italy) or in 0.4 mL sterile phosphate-buffered saline (PBS) depending on the subsequent analysis type. All the samples were immediately refrigerated and transported to the laboratory to be processed within 12 h.

### 4.4. Microbiological Analyses

The samples collected by RODAC plates were incubated using specific time and temperature conditions, depending on the microorganism type, as previously described [16,19,38,39]. At the end of the incubation time, the Colony Forming Units (CFUs) grown on plates were counted. *Staphylococcus* spp., including *S. aureus*, identification was performed on Baird–Parker medium and confirmed by API Staph (bioMerieux, Inc, Durham, NC, USA), as previously described [18,22]. In each sampling campaign, 960 samples were collected from the two hospitals; a total of 7680 samples were collected and analyzed in the whole study.

### 4.5. Molecular Analyses

The samples collected by swabs and put in TSB broth were incubated at 37 °C for 24 h to obtain a controlled microbial amplification. Then, the microbial suspension was collected by centrifugation (12,000× *g* for 5 min) and total DNA was extracted from the pelletized microbes by a commercial kit (Exgene Cell SV mini kit, Gene All, Seoul, South Korea), following the manufacturer’s instructions. A 1 µg sample of extracted DNA was then analyzed using the Microbial DNA qPCR Array for Antibiotic Resistance Genes (Qiagen, Hilden, Germany), allowing the detection and quantification of 84 AMR genes, as previously described [12,19]. Overall, 90 samples were analyzed for each hospital (180 total samples).

In parallel, swabs collected in PBS were directly frozen at −80 °C. The total nucleic acids (TNA) were then extracted by using a Maxwell CSC platform equipped with the HT Viral TNA Kit (Promega, Milan, Italy), following the manufacturer’s instructions, and 10 ng of TNA was analyzed for the quantification of Sb-1 bacteriophage load by a specific qPCR designed in the ORF79 (major capsid protein) gene of the *Staphylococcus* phage Sb-1, as previously described [38]. Overall, 240 environmental samples were collected from each hospital (480 total samples).

### 4.6. Antimicrobial and Phage Susceptibility Tests

All identified *S. aureus* isolates from collected samples in the pre-PCHS period (T0_1_ and T0_2_) were characterized for antibiotic susceptibility by the Kirby–Bauer disk diffusion test following the criteria outlined by the Clinical and Laboratory Standard Institute (CLSI). Zones of inhibition (expressed in mm) were measured, and the interpretation of results was based on the CLSI reference criteria [40].

Both *S. aureus* and coagulase negative staphylococci were further tested for their susceptibility to the available Sb-1 anti-staphylococcal phage preparation by spot test, as previously described [37,38].

### 4.7. Statistical Analyses

Statistical analyses were performed using the GraphPad Prism software. Parametric Student’s t-tests were used assuming as statistically significant a *p* value of at least < 0.05. To analyze the resistome data, the Bonferroni correction for multiple comparisons was applied to the value detected in Student’s t test, assuming a corrected *p_c_* value ≤ 0.05 as statistically significant.

## 5. Conclusions

Persistent microbial contamination in the hospital environment contributes to the onset of HAIs which are often sustained by pathogens characterized by high AMR. Chemical disinfection, massively used during the COVID-19 pandemic, acts only temporarily, and can promote the selection of AMR strains, thus potentially worsening this concern. In contrast, based on previous data, PCHS probiotic-based sanitation combined with targeted killing by bacteriophages (PCHSφ) may have the potential to provide quick and stable elimination of selected bacterial targets. Here we tested the applicability and effectiveness of such a combined sanitation in two large public Italian hospitals, showing that PCHSφ could efficiently remove bacterial targets including resistant ones. These data suggest that a biological sanitation approach may substantially ameliorate AMR and HAI concerns.

## Figures and Tables

**Table 1:** Emergency chlorine disinfections performed in enrolled wards (*).

Healthcare Center	Study Period		
	T0	T1	T2
HS-1	2	2	3
HS-2	2	6	17

(*) Results show the total number of 3% chlorine disinfections performed in the indicated study periods.

**Table 2:** Staphylococcal contamination in enrolled bathrooms (*).

Healthcare Center	Sanitation Type		
	Chemical	PCHS	PCHSφ
HS-1	17,053 CFU/m^2^ (100%)	10,949 CFU/m^2^ (−35.8%)	6473 CFU/m^2^ (−62.1%)
HS-2	14,948 CFU/m^2^ (100%)	16,316 CFU/m^2^ (+9%)	17,474 CFU/m^2^ (+16%)

(*) Results are expressed as mean values of staphylococcal CFU/m^2^ detected during T0, T1, and T2 periods. Percentages of reduction observed in T1 and T2 periods compared with T0 period values are also indicated in parentheses.

**Table 3:** Most prevalent R genes harbored by HS-1 and HS-2 microbiomes (*).

R gene/Species	Resistance to	Bacteria	Settings	
			HS-1	HS-2
*AAC (6)-Ib-cr*	Fluoroquinolones	*Enterobacteriaceae*	0.33	0
*AadA1*	Aminoglycosides	*Enterobacteriaceae*, *Staphylococcus*, *Streptococcus*	0.29	0
*AacC2*	Aminoglycosides	*Enterobacteriaceae*	0	0.16
*CTX-M-9 Group*	β-lactams (ESBL)	*Enterobacteriaceae*	0.34	0
*Per-1 group*	β-lactams	*Pseudomonas*	0	0.18
*SHV*	β-lactams (ESBL)	*Enterobacteriaceae*	0.33	0
*SHV (156G)*	β-lactams (ESBL)	*Enterobacteriaceae*	0.34	0
*SHV (238S240K)*	β-lactams (ESBL)	*Enterobacteriaceae*	0.34	0
*ACT 5/7 group*	β-lactams (ESBL)	*Enterobacteriaceae*	0	0.68
*OXA-23 Group*	β-lactams	*Acinetobacter*	0	1.18
*OXA-51 Group*	Carbapenems (carbapenemases)	Gram-negative	0	1.12
*QnrA*	Fluoroquinolones	Gram-negative	0.34	0
*QnrS*	Fluoroquinolones	Gram-negative	0	0.24
*ermA*	Macrolides (erythromycin)	*Staphylococcus*, *Streptococcus*	1.27	1.58
*ermB*	Macrolides (erythromycin)	*Staphylococcus*, *Streptococcus*	2.66	3.29
*ermC*	Macrolides (erythromycin)	*Staphylococcus*, *Streptococcus*	3.74	1.83
*mefA*	Macrolides (erythromycin)	*Staphylococcus*, *Streptococcus*, *Enterococcus*, *Clostridium*, *Bacteroides*	0.56	0.38
*msrA*	Macrolides (efflux pump)	*Staphylococcus*, *Streptococcus*, *Enterococcus*, *Pseudomonas*	4.21	2.95
*tetA*	Tetracyclin (efflux pump)	*Enterobacteriaceae*	0.41	0.16
*tetB*	Tetracyclin (efflux pump)	Gram-negative	0	1.41
*vanB*	Vancomycin	*Enterococcus*	0.25	0
*mecA*	Methicillin (β-lactamase)	*Staphylococcus*, *Enterococcus*	4.10	3.40
*S. aureus*	Bacterial species	*S. aureus*	0.78	0.99
*spa*	*S. aureus* virulence gene	*S. aureus*	0.14	0.40

(*) Results are expressed as Log_10_ Fold Change of R genes detected at T0 compared with negative control (NTC). Activity of R genes and most frequent species in which they are detected are also shown.

## Data Availability

All data supporting reported results are included in the manuscript.

## References

[1] Gilbert J.A., Stephens B. (2018). Microbiology of the built environment. Nat. Rev. Microbiol..

[2] Mahnert A., Moissl-Eichinger C., Zojer M., Bogumil D., Mizrahi I., Rattei T., Martinez J.L., Berg G. (2019). Man-made microbial resistances in built environments. Nat. Commun..

[3] National Academies of Sciences, Engineering and Medicine, National Academy of Engineering (2017). Microbiomes of the Built Environment: A research Agenda for Indoor Microbiology, Human Health, and Buildings.

[4] Allegranzi B., Bagheri Nejad S., Combescure C., Graafmans W., Attar H., Donaldson L., Pittet D. (2011). Burden of endemic health-care-associated infection in developing countries: Systematic review and meta-analysis. Lancet.

[5] ECDC (2013). Point Prevalence Survey of Healthcare-Associated Infections and Antimicrobial Use in European Acute Care Hospitals. https://www.ecdc.europa.eu/en/publications-data/point-prevalence-survey-healthcare-associated-infections-and-antimicrobial-use-0.

[6] ECDC (2015). European Surveillance of Healthcare Associated Infections in Intensive Care Units. http://ecdc.europa.eu/en/healthtopics/Healthcare-associated_infections.

[7] Kramer A., Schwebke I., Kampf G. (2006). How long do nosocomial pathogens persist on inanimate surfaces? A systematic review. BMC Infect. Dis..

[8] Pochtovyi A.A., Vasina D.V., Kustova D.D., Divisenko E.V., Kuznetsova N.A., Burgasova O.A., Kolobukhina L.V., Tkachuk A.P., Gushchin V.A., Gintsburg A.L. (2021). Contamination of hospital surfaces with bacterial pathogens under the current COVID-19 outbreak. Int. J. Environ. Res. Public Health.

[9] Avershina E., Shapovalova V., Shipulin G. (2021). Fighting antibiotic resistance in hospital-acquired infections: Current state and emerging technologies in disease prevention, diagnostics and therapy. Front. Microbiol..

[10] Marimuthu K., Pittet D., Harbarth S. (2014). The effect of improved hand hygiene on nosocomial mrsa control. Antimicrob. Resist. Infect. Control.

[11] Ghafoor D., Khan Z., Khan A., Ualiyeva D., Zaman N. (2021). Excessive use of disinfectants against COVID-19 posing a potential threat to living beings. Curr. Res. Toxicol..

[12] Vandini A., Temmerman R., Frabetti A., Caselli E., Antonioli P., Balboni P.G., Platano D., Branchini A., Mazzacane S. (2014). Hard surface biocontrol in hospitals using microbial-based cleaning products. PLoS ONE.

[13] D’Accolti M., Soffritti I., Bonfante F., Ricciardi W., Mazzacane S., Caselli E. (2021). Potential of an eco-sustainable probiotic-cleaning formulation in reducing infectivity of enveloped viruses. Viruses.

[14] Kampf G. (2018). Biocidal agents used for disinfection can enhance antibiotic resistance in gram-negative species. Antibiotics.

[15] Dai D., Prussin A.J., Marr L.C., Vikesland P.J., Edwards M.A., Pruden A. (2017). Factors shaping the human exposome in the built environment: Opportunities for engineering control. Environ. Sci. Technol..

[16] Li S., Yang Z., Hu D., Cao L., He Q. (2021). Understanding building-occupant-microbiome interactions toward healthy built environments: A review. Front. Environ. Sci. Eng..

[17] D’Accolti M., Soffritti I., Bini F., Mazziga E., Mazzacane S., Caselli E. (2022). Pathogen control in the built environment: A probiotic-based system as a remedy for the spread of antibiotic resistance. Microorganisms.

[18] Caselli E., Brusaferro S., Coccagna M., Arnoldo L., Berloco F., Antonioli P., Tarricone R., Pelissero G., Nola S., La Fauci V. (2018). Reducing healthcare-associated infections incidence by a probiotic-based sanitation system: A multicentre, prospective, intervention study. PLoS ONE.

[19] Caselli E., Arnoldo L., Rognoni C., D’Accolti M., Soffritti I., Lanzoni L., Bisi M., Volta A., Tarricone R., Brusaferro S. (2019). Impact of a probiotic-based hospital sanitation on antimicrobial resistance and hai-associated antimicrobial consumption and costs: A multicenter study. Infect. Drug Resist..

[20] Caselli E., D’Accolti M., Soffritti I., Lanzoni L., Bisi M., Volta A., Berloco F., Mazzacane S. (2019). An innovative strategy for the effective reduction of mdr pathogens from the nosocomial environment. Adv. Exp. Med. Biol..

[21] Caselli E., D’Accolti M., Soffritti I., Piffanelli M., Mazzacane S. (2018). Spread of mcr-1-driven colistin resistance on hospital surfaces, italy. Emerg. Infect. Dis..

[22] Caselli E., D’Accolti M., Vandini A., Lanzoni L., Camerada M.T., Coccagna M., Branchini A., Antonioli P., Balboni P.G., Di Luca D. (2016). Impact of a probiotic-based cleaning intervention on the microbiota ecosystem of the hospital surfaces: Focus on the resistome remodulation. PLoS ONE.

[23] D’Accolti M., Soffritti I., Mazzacane S., Caselli E. (2019). Fighting amr in the healthcare environment: Microbiome-based sanitation approaches and monitoring tools. Int. J. Mol. Sci..

[24] Evran S., Tayyarcan E.K., Acar-Soykut E., Boyaci I.H. (2022). Applications of bacteriophage cocktails to reduce salmonella contamination in poultry farms. Food Environ. Virol..

[25] Guenther S., Huwyler D., Richard S., Loessner M.J. (2009). Virulent bacteriophage for efficient biocontrol of listeria monocytogenes in ready-to-eat foods. Appl. Environ. Microbiol..

[26] Gutierrez D., Rodriguez-Rubio L., Martinez B., Rodriguez A., Garcia P. (2016). Bacteriophages as weapons against bacterial biofilms in the food industry. Front. Microbiol..

[27] Fan J., Zeng Z., Mai K., Yang Y., Feng J., Bai Y., Sun B., Xie Q., Tong Y., Ma J. (2016). Preliminary treatment of bovine mastitis caused by staphylococcus aureus, with trx-sa1, recombinant endolysin of s. Aureus bacteriophage ime-sa1. Vet. Microbiol..

[28] Goode D., Allen V.M., Barrow P.A. (2003). Reduction of experimental salmonella and campylobacter contamination of chicken skin by application of lytic bacteriophages. Appl. Environ. Microbiol..

[29] Gutierrez D., Fernandez L., Rodriguez A., Garcia P. (2019). Role of bacteriophages in the implementation of a sustainable dairy chain. Front. Microbiol..

[30] Jassim S.A., Limoges R.G., El-Cheikh H. (2016). Bacteriophage biocontrol in wastewater treatment. World J. Microbiol. Biotechnol..

[31] Moye Z.D., Woolston J., Sulakvelidze A. (2018). Bacteriophage applications for food production and processing. Viruses.

[32] Nakai T., Park S.C. (2002). Bacteriophage therapy of infectious diseases in aquaculture. Res. Microbiol..

[33] O’Sullivan L., Bolton D., McAuliffe O., Coffey A. (2019). Bacteriophages in food applications: From foe to friend. Annu. Rev. Food Sci. Technol..

[34] Rahimzadeh G., Zazouli M.A., Rezai M.S. (2022). Potential of lytic bacteriophages as disinfectant to control of pseudomonas aeruginosa on fomites. J. Environ. Health Sci. Eng..

[35] Jensen K.C., Hair B.B., Wienclaw T.M., Murdock M.H., Hatch J.B., Trent A.T., White T.D., Haskell K.J., Berges B.K. (2015). Isolation and host range of bacteriophage with lytic activity against methicillin-resistant staphylococcus aureus and potential use as a fomite decontaminant. PLoS ONE.

[36] Chen L.K., Chang J.C., Chu H.T., Chen Y.T., Jiang H.L., Wang L.S., Teh S.H., Yang H.H., Chen D.S., Li Y.Z. (2022). Preoptimized phage cocktail for use in aerosols against nosocomial transmission of carbapenem-resistant acinetobacter baumannii: A 3-year prospective intervention study. Ecotoxicol. Environ. Saf..

[37] Chen L.K., Liu Y.L., Hu A., Chang K.C., Lin N.T., Lai M.J., Tseng C.C. (2013). Potential of bacteriophage phiab2 as an environmental biocontrol agent for the control of multidrug-resistant acinetobacter baumannii. BMC Microbiol..

[38] D’Accolti M., Soffritti I., Piffanelli M., Bisi M., Mazzacane S., Caselli E. (2018). Efficient removal of hospital pathogens from hard surfaces by a combined use of bacteriophages and probiotics: Potential as sanitizing agents. Infect. Drug Resist..

[39] D’Accolti M., Soffritti I., Lanzoni L., Bisi M., Volta A., Mazzacane S., Caselli E. (2019). Effective elimination of staphylococcal contamination from hospital surfaces by a bacteriophage-probiotic sanitation strategy: A monocentric study. Microb. Biotechnol..

[40] Curran E.T., Wilkinson M., Bradley T. (2019). Chemical disinfectants: Controversies regarding their use in low risk healthcare environments (part 1). J. Infect. Prev..

[41] Nabi G., Wang Y., Hao Y., Khan S., Wu Y., Li D. (2020). Massive use of disinfectants against COVID-19 poses potential risks to urban wildlife. Environ. Res..

[42] Zeshan B., Karobari M.I., Afzal N., Siddiq A., Basha S., Basheer S.N., Peeran S.W., Mustafa M., Daud N.H.A., Ahmed N. (2021). The usage of antibiotics by COVID-19 patients with comorbidities: The risk of increased antimicrobial resistance. Antibiotics.

[43] Lai C.C., Chen S.Y., Ko W.C., Hsueh P.R. (2021). Increased antimicrobial resistance during the COVID-19 pandemic. Int. J. Antimicrob. Agents.

[44] Clancy C.J., Buehrle D.J., Nguyen M.H. (2020). Pro: The COVID-19 pandemic will result in increased antimicrobial resistance rates. JAC Antimicrob. Resist..

[45] WHO Tackling Antimicrobial Resistance in the COVID-19 Pandemic. https://www.who.int/bulletin/volumes/98/7/20-268573.pdf.

[46] Tarricone R., Rognoni C., Arnoldo L., Mazzacane S., Caselli E. (2020). A probiotic-based sanitation system for the reduction of healthcare associated infections and antimicrobial resistances: A budget impact analysis. Pathogens.

[47] D’Accolti M., Soffritti I., Mazzacane S., Caselli E. (2021). Bacteriophages as a potential 360-degree pathogen control strategy. Microorganisms.

[48] D’Accolti M., Soffritti I., Bini F., Mazziga E., Cason C., Comar M., Volta A., Bisi M., Fumagalli D., Mazzacane S. (2023). Shaping the subway microbiome through probiotic-based sanitation during the COVID-19 emergency: A pre–post case–control study. Microbiome.

[49] Soffritti I., D’Accolti M., Cason C., Lanzoni L., Bisi M., Volta A., Campisciano G., Mazzacane S., Bini F., Mazziga E. (2022). Introduction of probiotic-based sanitation in the emergency ward of a children’s hospital during the COVID-19 pandemic. Infect. Drug Resist..

[50] Chen X., Liu Y., Chai S., Guo J., Wu W. (2018). Inactivation of lactobacillus virulent bacteriophage by thermal and chemical treatments. J. Food Prot..

[51] Ge Y., Zhang X., Shu L., Yang X. (2021). Kinetics and mechanisms of virus inactivation by chlorine dioxide in water treatment: A review. Bull. Environ. Contam. Toxicol..

[52] ISS (2020). Interim recommendations on cleaning and disinfection of non-healthcare settings during COVID-19 health emergency: Surfaces, indoor environments and clothing. ISS COVID-19 Working Group on Biocides, ISS ed..

